# Abduction treatment in stable hip dysplasia does not alter the acetabular growth: results of a randomized clinical trial

**DOI:** 10.1038/s41598-020-66634-1

**Published:** 2020-06-15

**Authors:** V. Pollet, R. M. Castelein, M. van de Sande, M. Witbreuk, A. K. Mostert, A. Besselaar, C. van Bergen, E. Beek, C. S. P. M. Uiterwaal, R. J. B. Sakkers

**Affiliations:** 1 Royal Manchester Children’s hospital, Department of Pediatric Orthopedics and Traumatology, Manchester, United Kingdom; 2 University Medical Center Utrecht, Department of Orthopedics, Utrecht, The Netherlands; 3 Leiden University Medical Center, Leiden, The Netherlands; 4 Amsterdam Medical University Center, Amsterdam, The Netherlands; 5 Isala Hospital, Zwolle, The Netherlands; 6 Maxima Medical Center, Eindhoven, The Netherlands; 7 University Medical Center Utrecht, Department of Radiology, Utrecht, The Netherlands; 8 University Medical Center Utrecht, Julius Center for Health Sciences and Primary Care, Utrecht, The Netherlands

**Keywords:** Medical research, Paediatric research, Bone

## Abstract

**Background** The effect of bracing over natural history of stable dysplastic hips is not well known. This multicenter randomized trial aimed at objectifying the effect of abduction treatment versus active surveillance in infants of 3 to 4 months of age. **Methods** Patients were randomized to either Pavlik harness or active surveillance group. Ultrasound was repeated at 6 and 12 weeks post randomization. The primary outcome was the degree of dysplasia using the Graf α-angle at 6 months of age. The measurement of the acetabular index (AI) on plain pelvis X-rays was used to identify persistent dysplasia after 9 months and walking age (after 18 months). **Findings** The Pavlik harness group (n = 55) and active surveillance group (n = 49) were comparable for predictors of outcome. At 12 weeks follow-up the mean α-angle was 60.5° ± 3.8° in the Pavlik harness group and 60.0° ± 5.6° in the active surveillance group. (p = 0.30). Analysis of secondary outcomes (standard of care) showed no treatment differences for acetabular index at age 10 months (p = 0.82) and walking age (p = 0.35). **Interpretation** Pavlik harness treatment of stable but sonographic dysplastic hips has no effect on acetabular development. Eighty percent of the patients will have a normal development of the hip after twelve weeks. Therefore, we recommend observation rather than treatment for stable dysplastic hips.

## Introduction

Developmental Dysplasia of the Hip or DDH is one of the most common pediatric orthopedic problems with an incidence varying from 1–6/1000 depending on regional predisposition and ethnic differences^1^. Hip dysplasia in the first months of life is clinically best detected by instability or dislocation of the hip. The Galeazzi test shows the leg length discrepancy due to the dislocated femoral head and the Ortolani maneuver will be positive when a hip is dislocated and can be reduced. In case of instability Barlow and Ortolani maneuvers will displace and relocate the hip joint^2^. Before the introduction of ultrasound, radiologic evaluation was used to assess acetabular development^3^. However, radiographic measurements are considered inaccurate below the age of 6 months due to a rather large variation in normal bone maturation^3^.

The introduction of sonographic imaging of the infant hip gave rise to definitions of instability and a classification of different types of severities of dysplasia of the acetabulum^4,5^. In the early 1980’s, Graf and Harcke published their observations and definitions. In 1993, Graf and Harcke reached a consensus that a standard examination could be accomplished by two orthogonal views (one coronal and one sagittal) and one view should also include a stress test. The latter was used to differentiate between stable and unstable hips^6^. Others contributed to the development of US examinations such as Morin describing the percentage of Femoral Head Coverage (FHC), which was modified by Terjesen, to quantify the hip coverage, with less than 50% defined as DDH^7–9^.

Currently, abduction treatment, preferably started in the first months of life, is viewed as the standard of care for all types of hip dysplasia. There exists, however, a considerable geographic variation in consistency of diagnostic criteria for DDH^10^. While in some countries, clinical findings and/or risk factors will determine the need for ultrasound hip screening, in other countries all newborns are screened for DDH and receive early treatment. Some have questioned the latter as potential for over-diagnosing and therefore unnecessary treatment, as 85% of infantile DDH will resolve spontaneously by the age of 3 months^11^. The most commonly used abduction brace is the Pavlik harness. The outcome of this treatment is widely considered successful if started at a young age (less than 6 months) and in cases where the hip is not rigidly dislocated^12^. However, when studying Pavlik’s original publication, the device was designed to gradually and a-traumatically reduce an unstable or dislocated hip, not for treatment of a dysplastic hip that is well centered and stable inside the acetabulum. The use of dynamic, non-rigid stirrups aim at decreasing the chances of avascular necrosis often seen in alternative rigid immobilization^13^. Until now, comparative studies in treatment of stable DDH starting treatment at 2 weeks and at 6 weeks did not show a difference in outcome nor did a randomized trial comparing treatment versus no treatment between 6 weeks and 3 months^14–17^. The question therefore arises if well-centered stable hips that are classified as DDH by Graf (Type IIb/IIc hips) are a true pathology or merely hips within the normal spectrum of hip development. If the latter is true, what is the effect of abduction treatment on the development of well-centered stable hips?

This randomized multi-center study was designed to investigate if abduction treatment, for the duration of 12 weeks, alters the sonographic development of well-centered stable hips confirmed by ultrasound at the age between 3 and 4 months.

## Methods

### Participants

After Ethics board approval (08/084 - University Medical Center Utrecht, The Netherlands) of the five participating hospitals (UMC Utrecht, Leiden UMC, Amsterdam Medical University Center, Isala Hospital Zwolle, Maxima Medical Center Eindhoven) all patients between 3 and 4 months of age diagnosed with clinically stable hip dysplasia according to Graf’s classification i.e. Graf type IIb and type IIc were included in the study. Calendar age was corrected for premature birth by subtracting the number of weeks prior to full term pregnancy (i.e. 38 weeks) from the calendar age. Patients with co-morbidity such as congenital deformities, previous treatment, hip instability or lack of consent were excluded from the study. Parents of eligible infants were given 7 days to consider participation in this study and a singed consent was obtained. This study was performed according to the Statement on Helsinki guidelines of 2008.

### Procedures

A single independent investigator (VP), who was not involved in the treatment of the patients, randomly allocated participants to either Pavlik harness treatment versus active surveillance group by computer-generated randomization in strata for type of dysplasia and participating hospital. Pavlik harness treatment was started within one week. Parents were shown how to apply/remove the harness as they were allowed to remove the harness for bathing. The active surveillance group was reviewed in clinic after 6 weeks. Parents were free to alter the treatment or request no treatment at any time for the duration of the study. All available data prior to this decision were included in the analysis.

All patients were seen in clinic with ultrasound of the hips at 6 weeks and at 12 weeks follow-up. The bony roof angle (alpha angle, α °) and Graf classification at 12 weeks follow-up were noted as primary outcome. A senior pediatric radiologist (EB) read all measurements blinded for study intervention. Applying Graf’s eligibility criteria for hip ultrasound, the best image of 3 ultrasound scans was assessed to measure the alpha angle. For the active surveillance group, lack of improvement of the alpha angle and/or instability at 6 or 12 weeks ultrasound scan required treatment with Pavlik harness. Complications such as femoral nerve palsy and progression to a dislocated hip causing cessation of the Pavlik harness were noted in the medical records as safety outcome.

As standard of care in The Netherlands, X-rays of the pelvis with measurement of Acetabular Index angle (AI) are routinely taken 3 months after the 6 months ultrasound (around 9 months of age) and at least after 2 years of age (i.e. after walking age). As secondary outcome, the AI measurements were graded according to the modified Tönnis classification for residual dysplasia^4^.

### Statistical analysis

The a priori hypothesis in the study protocol was that there is no difference in treatment effect between the two groups. A Two One-sided T- test (TOTS) of Equivalence showed that 50 children were needed in each group to reach a power of 90% with a significance of 5% (two-sided) and average alpha angle of 58 degrees in both groups (SD 8.2). The mean group difference ranging between −5 and + 5 degrees led to a conclusion of Equivalence. In case of bilateral stable hip dysplasia, the average of the alpha-angles was used.

For the primary outcome analysis, linear regression analysis at primary endpoint (i.e. alpha angle at 12 weeks follow-up) was used as the dependent variable and the treatment group indicator as independent variable to calculate the mean difference in alpha angle with a 95% confidence interval.

For secondary outcome analysis, as part of standard of care, Fisher exact test analysis of the Acetabular Index was used to show significance, with a p-value of less than 0.05.

## Results

**Figure 1:**
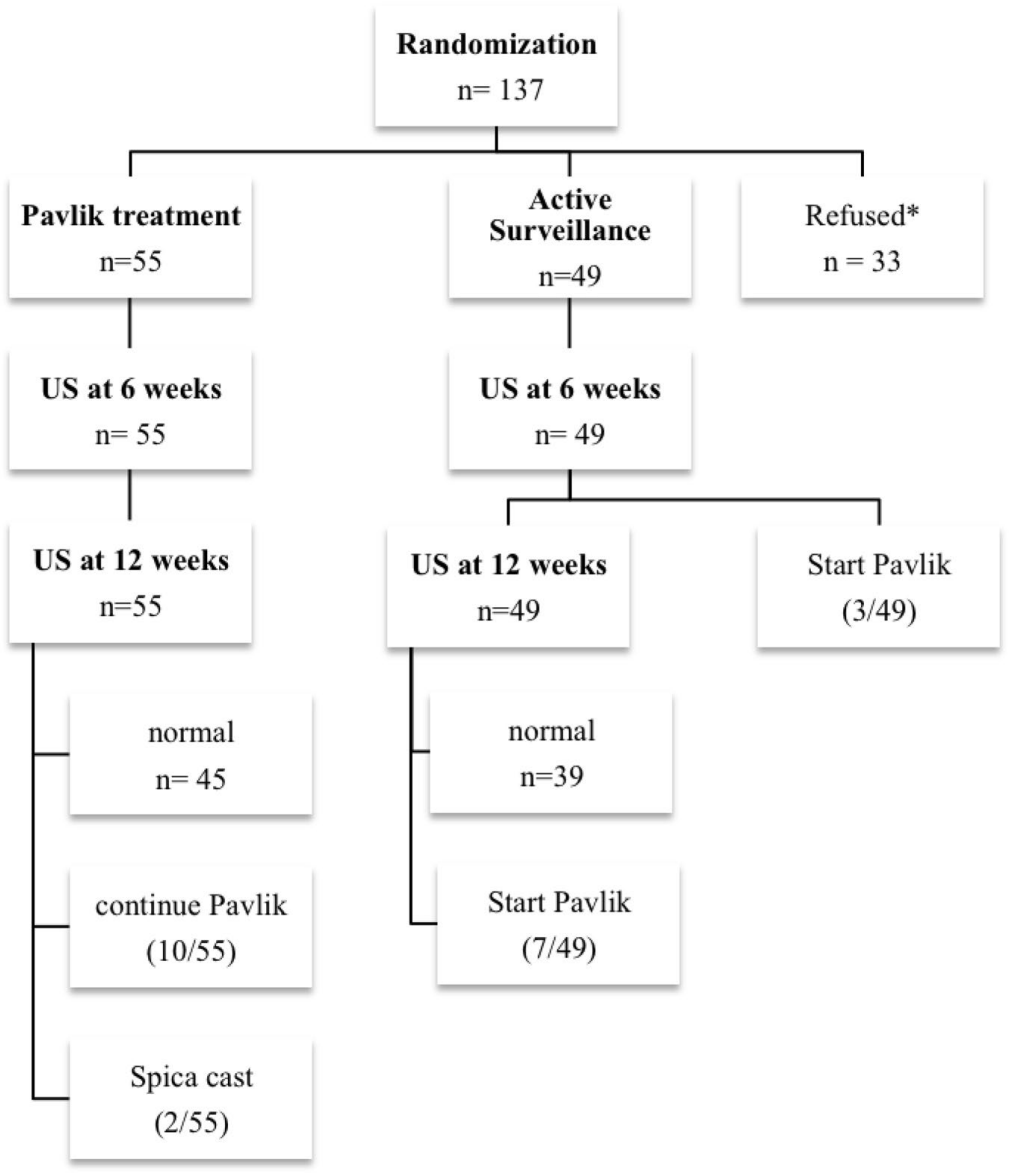
Study flow diagram. *After randomization, 33 hips were excluded after parents refused the allocated treatment plan. The included 104 hips were assessed by Intention-To-Treat analysis.

Between 2009 and 2015, parents of 137 patients, meeting the inclusion criteria, gave preliminary consent. After randomization, the consent was withdrawn in 33 patients since parents decided to alter the allocated treatment (18 Pavlik harness treatment versus 15 active surveillance). One hundred and four patients remained for participation in this study. Fifty-five patients were allocated in the Pavlik harness treatment group and 49 patients to the active surveillance group. (Fig. 1).

After 6 weeks of observation, 3 patients received a Pavlik harness in the active surveillance group because of deterioration of the alpha angle. Another 7 patients were treated after 12 weeks of observation due to persistent dysplasia (Graf IIb). Thirty-nine hips (79.6%) normalized after 3 months of active surveillance. After 12 weeks of Pavlik harness treatment, the harness was continued or switched to an abduction brace due to residual dysplasia on ultrasound in 10 patients (18.2%). Despite the prolonged treatment, two patients developed instability and were subsequently treated with closed reduction and spica cast. There were no issues with compliance and femoral nerve palsy in the Pavlik harness treated patients.

At treatment initiation, randomly allocated groups were comparable for bony roof angle, age, gender, affected side and risk factors such as breech position, positive family history and first child. (Table 1).

**Table 1:** Patients characteristics, bony roof angles (α-angle) and important predictors of outcome are comparable for both groups.

	Pavlik harness n = 55	Active Surveillance n = 49
age (weeks) (SD)	14.3 (1.8)	14.1 (2.1)
**gender (n,%)**		
female	50 (91)	43 (88)
male	5 (9)	6 (12)
**side of dysplasia**		
right hip	13	14
left hip	37	30
bilateral	5	5
affected hip α-angle (degrees)(SD)	54.2 (3.3)	55.0 (2.8)
**Graf classification (n,%)**		
mild	50 (91)	47 (96)
severe	5 (9)	2 (4)
breech (n,%)	13 (24)	14 (28)
+ fam history (n,%)	22 (40)	14 (28)
1st child (n,%)	14 (25)	10 (20)
twins (n,%)	0 (0)	1 (2) (Breech)

Characteristics of the group that withdrew after randomization were comparable to the inclusion group. Since their outcome data were not according to protocol, they were excluded from the analyses. As this may have affected the randomization, we added an analysis that was fully adjusted for covariates. All but 7 children, had Graf type IIb hips with an average α-angle of 54.2° ± 3.3° for the treated group and 55° ± 2.8° for the active surveillance group. The progression of alpha angle was calculated for both the treatment group as the active surveillance group at 6 weeks and 12 weeks. There was no difference in treatment effect between the two groups. The alpha angle corrected over time in both groups at a similar rate. (Table 2).

**Table 2:** Bony roof angle (α) improvement over the observed period of 12 weeks. Adjusted difference: for age, gender and measurement 1. For children with bilateral affected hips, left and right angle measurements were averaged.

affected hip α (°)(SD)	Treatment				
	**Pavlik harness treatment**	**Active Surveillance**	adjusted difference	95%CI	p-value
at 6 weeks	58.8 (±5.5)	58.0 (±5.2)	−1.39	−3.46,0.67	0.18
at 12 weeks	60.5(±3.8)	60.0 (±5.6)	−1.00	−2.92,0.91	0.30

As part of standard of care, we were able to identify 90 out of the 104 (86.5%) patients who received a pelvis X-ray imaging on average 3 months after ultrasound: 50 patients (= 54 hips) in the treatment group and 40 patients (= 41 hips) in the active surveillance group at 10.4 ± 4.4 months and 10.2 ± 3.2 months respectively. There was no difference in AI between the two groups. (p = 0.82) (Table 3).

**Table 3:** Modified Tönnis classification for degree of dysplasia (acetabular index) on pelvis X-rays at minimal 3 months after 2nd US measurement and after walking age. AI at 10 months: normal ≤ 30 °; 30° < mild dysplasia >35°; Severe dysplasia ≥ 35°. AI at 2 years: normal ≤ 25 °, 25°< mild dysplasia >30°, severe dysplasia ≥ 30°.

Modified Tönnis classification		Pavlik harness treatment	Active Surveillance	p-value
10 months - average AI		26.4° ± 4.6° (range 19°–44°)	26.2° ± 5.0° (range: 16–37°)	0.82
	normal(n,%)	40 (80)	30 (75)	
	mild (n,%)	8 (16)	8 (20)	
	severe (n,%)	2 (4)	2 (5)	
2 years - average AI		22.9° ± 5.1°(range 8°–33°)	23.0° ± 4.4° (range 15° −30°)	0.35
	normal (n,%)	24 (60)	18 (58)	
	mild (n,%)	13 (33)	12 (39)	
	severe (n,%)	3 (7)	1 (3)	

Furthermore, in 71 (68%) patients the hips were imaged after walking age. Forty patients of the treatment group had a latest follow-up at 30 ± 16 months. The latest follow-up for the watchful waiting group was the same at 30 ± 12.5 months. Again, there was no difference in residual dysplasia between the two groups. (p = 0.35).

## Discussion

This multicenter randomized-controlled study did not show differences in outcome of treatment with abduction bracing versus active surveillance in infants of 3 to 4 months of age with sonographic dysplastic but well-centered stable hips. To our knowledge, no previous trials studied the effect of abduction treatment for stable hip dysplasia beyond 3 months of age. Rozendahl and colleagues and Wood and colleagues examined the outcome of splinting versus observation in newborns during the first month of life^14,18^. (Table 4).

**Table 4:** Similar results in comparative studies of treatment versus sonographic surveillance.

Authors	Wood *et al*. (FHC%) retrospective n = 44	Rosendahl *et al*. (α-angle) randomized n = 128	This study (α-angle) randomized n = 104
age at diagnosis	2–6 weeks	1-2 days	3–4 months
**affected hip**			
treatment	36.7 (%)	47°	54.2°
surveillance	32.8 (%)	47°	55°
**at 6 weeks follow-up**			
treatment	54.3 (%)	58°	58.8°
surveillance	48.6(%)	55°	58°
**at 12 weeks follow-up**			
treatment	24.7°(AI)	61°	60.5°
surveillance	24.2°(AI)	59°	60°
**10–12 months of age (AI)**			
treatment	23.5°	24.2°	24.4°
surveillance	21.6°	24.2°	26.2°
**after walking age (AI)**			
treatment	n/a	n/a	22.9°
surveillance	n/a	n/a	23°

FHC% = Femoral Head Coverage - normal FHC ≥ 40%; AI = Acetabular index (°).

Wood and colleagues, examined prospectively the outcome of splinting versus observation in infants between 2 to 6 weeks of age, with stable but dysplastic hips, defined as Morin’s ratio of femoral head to acetabular diameter of <40% and less than 2 mm displacement from the floor of the acetabulum during Barlow’s manoeuver. More than 2 mm displacement was considered unstable and those hips were excluded. Mean acetabular coverage was 32.8% and 36.7 respectively with 54.3% after 3 months of splinting compared to 48.5% in the non-splinted group. Although this was statistically significant, the absolute percentage difference of 5.8% is not clinically relevant as both are values of normalized hips (>40%). Furthermore, the acetabular indices on radiographs after 3 months (24.8° vs 24.3°) and 24 months (21.6° vs 23.5°) did not show any difference. The authors concluded that abduction treatment has no lasting benefit and therefore recommended follow-up until the age of 3 months for stable well-centered hips with sonographic DDH rather to avoid unnecessary treatment. Our results show a similar continuation of acetabular development with or without treatment beyond the age of 3 months for stable well-centered hips with sonographic DDH (Graf type IIb/IIc). Wilkinson *et al*. investigated the natural history of α-angles in relation to age, gender and side and found an average of 5.0° (range 4.4°–5.3°) of improvement during the first 3 months of life in normal hips. There was a slower increase of the alpha angles in the female patients and for the left hip^19^. In our study, we found a similar ongoing average improvement of untreated well-centered hips with sonographic DDH of 5.0° over a period of 12 weeks. Treatment with a Pavlik harness did not accelerate the hip joint development. (p = 0.30).

All but 7 children had Graf type IIb hips. The two patients with IIc hips (α-angles of 46° and 48°) that were randomized in the active surveillance group did not show residual dysplasia after 12 weeks of observation. It would be of interest to confirm this in a larger population study of severe dysplastic hips. In comparison, Rozendahl and colleagues, conducted a randomized controlled trial of 128 newborns with stable dysplastic hips (α-angle between 43°–49°) (Graf type IIc or mild dysplasia according to Rozendahl’s modified Graf’s classification) with AI at 12 months on X-rays as primary outcome^18^. Half of the children in the surveillance group received treatment during the observation period of 6 months because of persistent dysplasia on ultrasound i.e. α-angle less than 50° after 6 weeks or less than 55° at 3 months follow-up. There was no increase in treatment duration due to the surveillance. Both groups showed similar results at one year follow-up (AI of 24.2° in both groups). The authors concluded that active sonographic surveillance halved the number of children requiring treatment with important implications for families and health care costs. In our study, a decrease or cease in progression of the α-angle led to treatment in only 3 patients (6.1%) during the active surveillance period of 3 months. After 12 weeks of follow-up, another 7 patients were treated with the Pavlik harness due to persistent Graf IIb dysplasia. In total, 10 out of 49 patients (20.4%) in the active surveillance group received treatment. This is in accordance to our recently published findings on the natural history of sonographic stable hips under six months of age where more than 80% will normalize without treatment^20^. This implies that even less patients will need treatment and the sonographic surveillance period can be extended until 6 months of age for Graf type IIb hips. Furthermore, this questions the sensitivity of ultrasonography distinguishing between true pathological hip morphology and normal ongoing development in stable hips. The current classifications based merely on bony-roof angles and instability testing aren’t able to identify those hips that will do poorly later in life and leads to overtreatment.

While not all patients received further follow-up as standard of care, analysis of the rate of further acetabular growth by measuring the acetabular indices showed no added effect of treatment even after more than 2 years. We believe this is the only study, in which patients with stable hip dysplasia are randomized for treatment or observation, that describes acetabular development beyond walking age. Pruszczynski and colleagues studied the natural history of acetabular growth in 48 hips with neonatal instability without dislocation/subluxation on ultrasound, i.e. reduced in rest and no dislocation on Barlow^21^. Acetabular indices progressively normalized (i.e AI ≤ 25°) in 100% of the cases at 3 years of age. Interestingly, they identified two groups: one with normal hips at 7 months, and a second group that normalizes after 7 months with 81% being normal by 24 months of age. The latter was significantly correlated with breech position and caesarean delivery. We also found the same percentage of normal hips (AI ≤ 25°) at 2 y follow-up, 80% and 75% respectively. At 30 months, 2 hips were still severely dysplastic according to the Tonnis criteria (AI ≥ 30°) and will need further follow-up.

Despite Pavlik harness treatment started at 3 to 4 months of age, two hips developed instability. Both patients were successfully treated with closed reduction and spica casting for 3 months and did not present residual dysplasia at walking age. Sibinski and colleagues analyzed long-term results of abduction treatment for Graf type IIb hips^22^. After 9 years, they found 20% to have residual dysplasia despite 66% of this subgroup having normal ultrasounds after treatment as an infant. Furthermore, Gardiner and colleagues, confirmed that some ultrasonographic unstable hips can be mistaken for normal on Graf’s static exam and therefor progress to instability during follow-up. Reversely, a Graf III hip can be stable on Harcke dynamic examination^23^. This could explain why two hips progressed further despite treatment, as they could have been more severely dysplastic than initially diagnosed on ultrasound.

The findings in this study make us reflect on the usefulness of current ultrasound classifications for stable hip dysplasia as they are not able to distinguish between normal developing hips and true pathologic hip dysplasia. Since the majority of hips that are classified as sonographic stable dysplastic hips show spontaneous normalization, more specific methods and definitions are needed to distinguish between normal developing hips and true hip dysplasia. Until we have better methods for the diagnosis of true hip dysplasia, we recommend observation, rather than treatment, of all well-centered stable hips according to the current ultrasound classifications. This would also avoid significant overtreatment (80%) with a burden to both the families and health care systems. Identifying true DDH cases will show insufficient improvement in time and might need some form of treatment at follow-up.

## Conclusion

Pavlik harness treatment in 55 three to four months old infants with well-centered, stable but dysplastic hips on ultrasound showed no difference compared to active surveillance in 49 infants with identical hip dysplasia after 12 weeks of observation. Furthermore, treatment with Pavlik harness did not accelerate the improvement of the bony roof angle (α-angle). To avoid overtreatment, observation of well-centered sonographic stable hips up to the age of 6 months seems sufficient in order to identify those hips that fail to improve spontaneously.
